# Insular functional connectivity in migraine with aura

**DOI:** 10.1186/s10194-022-01473-1

**Published:** 2022-08-19

**Authors:** Cédric Gollion, Fleur Lerebours, Federico Nemmi, Germain Arribarat, Fabrice Bonneville, Vincent Larrue, Patrice Péran

**Affiliations:** 1 Department of Neurology, University Hospital of Toulouse, 31059 cedex 9 Toulouse, France; 2 Toulouse NeuroImaging Center, ToNIC, University of Toulouse III, Inserm, Toulouse, France; 3 Department of Neuroradiology, University Hospital of Toulouse, Toulouse, France

**Keywords:** MRI, Functional MRI, Insula, Migraine, Migraine with aura, Cerebellum vermis

## Abstract

**Introduction:**

Insula plays an integrating role in sensory, affective, emotional, cognitive and autonomic functions in migraine, especially in migraine with aura (MA). Insula is functionally divided into 3 subregions, the dorsoanterior, the ventroanterior and the posterior insula respectively related to cognition, emotion, and somatosensory functions. This study aimed at investigating functional connectivity of insula subregions in MA.

**Methods:**

Twenty-one interictal patients with MA were compared to 18 healthy controls (HC) and 12 interictal patients with migraine without aura (MO) and were scanned with functional MRI during the resting state. Functional coupling of the insula was comprehensively tested with 12 seeds located in the right and left, dorsal, middle, ventral, anterior and posterior insula, by using a seed-to-voxel analysis.

**Results:**

Seed-to-voxel analysis revealed, in MA, a strong functional coupling of the right and left antero-dorsal insula with clusters located in the upper cerebellum. The overlap of these cerebellar clusters corresponded to the vermis VI. These functional couplings were not correlated to duration of MA, frequency of MA attacks nor time since last MA attack, and were not found in MO.

**Discussion:**

The anterior insula and superior cerebellum, including vermis VI, are components of the central Autonomic Nervous System (ANS) network. As these regions are involved in the control of cardiovascular parasympathetic tone, we hypothesize that this connectivity may reflect the cardiovascular features of MA.

**Conclusion:**

The anterior dorsal insula is connected with vermis VI in MA patients in the resting state. This connectivity may reflect the cardiovascular features of MA.

**Trial registration:**

NCT02708797.

## Introduction

Migraine is a disabling disorder affecting up to 15% of the global population, and is the second leading cause of years lived with disability [1, 2]. Migraine pain is related to the involvement of the trigeminovascular system [3], but migraine is not only a pain, as it is also accompanied by several sensory, autonomic, affective and cognitive disorders. These symptoms are posited to result from multiple brain networks involvement within the brainstem, the subcortical and cortical areas, beyond the trigemino-vascular system [4]. In about one third of migraine patients, migraine attacks are accompanied by an aura which is a transient progressive and fully reversible central neurological symptom, most often visual, occuring before the headache. The insula is involved in multiple cerebral functions such as sensorymotor processing, pain, taste, interoception, autonomic control, emotions, attention or salience which refers to the ability to select the most relevant information among multiple internal and external stimuli [5]. Parcellation of insula resulted in two architectonic subdivisions (posterior granular area and anterior dysgranular area) to thirteen multi-modal MRI subdivisions [5, 6]. Nevertheless, data-driven meta-analysis of human functional imaging studies supported a tripartite subdivision of the insula into a ventroanterior area, a dorsoanterior area and a posterior area. The ventroanterior insula is functionally coupled with limbic areas and is associated with emotion, chemosensation and autonomic functions. The dorsoanterior insula is connected to the anterior cingulate cortex and the dorsolateral prefrontal cortex and plays a role in cognitive tasks and executive control. Conversely, the posterior insula is connected to the somatosensory cortex and the suplementary motor area resulting in pain and somatosensory functions involvement [7]. Evidences support the involvement of the insula in several features of migraine pathophysiology from the ictal phase to chronicisation. During the ictal phase of spontaneous migraine attacks, Positron Emission Tomography (PET) studies revealed activation of bilateral insula cortex as well as other cortical areas, brainstem and diencephalic nuclei [8, 9]. In addition, functional MRI studies during the ictal phase showed a stronger activation of the anterior insula in response to olfactory stimulations, but a decreased functional connectivity (FC) of the anterior insula with the medial prefrontal cortex within the Default Mode Network (DMN) inversely proportional to the pain intensity [10, 11]. Another study showed a higher FC between the right thalamus and the left insular cortex during spontaneous migraine attacks [12]. During the intercital phase of migraine without aura (MO), the right posterior insula was identified as a hub of FC more strongly connected to the supplementary motor cortex and the paracentral lobule among other brain areas [13]. In high frequency migraine, defined by 8 to 14 monthly migraine days, compared to low frequency migraine, heat painful stimulations of the hand induced lower controlateral anterior insula and bilateral inferior insula activations, but a higher connectivity of bilateral insula with the left post central gyrus [14]. In chronic migraine, number of years of chronic migraine were correlated to the resting state FC between bilateral anterior insula and the right mediodorsal thalamus, as well as to the FC between the right anterior insula and the periaqueductal grey matter (PAG) [15]. Overall, the insula is posited to play a key role in migraine, acting as a « hub» of integration of autonomic, sensory, affective and cognitive functions [16]. Insula in migraine with aura (MA) is of specific interest as previous studies have found specific alterations of insular connectivity in MA. The anterior insula had a reduced connectivity with occipital areas in MA compared to MO and Healthy Controls (HC), and the connectivity changes between the left anterior insula and occipital areas were negatively correlated with headache severity in MA only [17]. In a study investigating cognitive functions in migraine and assessing the DMN, patients with MA presented an increased FC between the right insular cortex, the left angular gyrus, the left supramarginal gyrus, the right precentral gyrus and the right postcentral gyrus compared to MO. In patients with complex MA, defined by more than visual symptoms, the right anterior insula was more strongly connected within the sensorimotor network compared to simple visual aura and MO, and this increased FC could discriminate between complex MA and simple visual aura [18]. Moreover, in a PET/MRI brain study, uptake of [11C]PBR28, a glial activation maker, in the right posterior insula was correlated to the number of MA attacks [19]. However, this result was not compared to MO.These observations suggested that the insula exhibited altered connectivity in MA, however these studies have not taken into account the functional division of the insula. In fact, the studies have focused either on the salience network, which includes the anterior insula [17, 20, 21], or on the somato-sensory network, which includes the posterior insula [18] or to a few regions of interest that did not explore the insula in its subdivisions. To our knowledge, the FC of the insula’s functional subdivisions has not yet been comprehensively studied in MA. Therefore, in the present study, we aimed at investigating the bilateral insular connectivity in MA using seeds in the anterior, posterior, dorsal, middle and ventral insula.

## Methods

### Design and population

This study was retrospectively conducted from images acquired in a previous MRI protocol (Trial registration: NCT02708797). Twenty-three patients with MA patients aged 30 to 55 without history of neurological disease were compared to 23 age and sex matched HC. Volunteers were excluded in case of abnormal neurological examination or abnormal MRI. Diagnosis of MA was confirmed by a trained neurologist according to the ICHD-3 criteria [22]. Patients with MA were included during a pain-free period for at least 8 days. Age at migraine onset and aura onset, frequency of migraine attacks in the past twelve months, type of aura (visual, sensory, dysphasic or other), frequency of aura among all migraine attacks, time since last migraine attack, and preventive treatment were recorded. To appraise the specificity of results found in patients with MA, a post-hoc analysis was conducted in twelve patients with episodic MO from another study previously conducted in our center. This previous study compared the brain FC of patients with chronic migraine to patients with episodic migraine [23]. The fMRI protocol was the same and the patients with MO were scanned during a pain-free period of at least 72 h.

### Ethics

The study was approved by the local institutional Ethics Committee (Comité de protection des personnes Sud-Ouest I). All participants gave written informed consent.

### Images acquisition

MRI images were acquired on a 3 T MR imager (Philips Achieva dStream 3 T 32-channel coils). All MRI were interpreted by a senior neuroradiologist. All volunteers were evaluated during the resting state, awake and eyes closed. No activation task was performed. For the resting-state functional MRI (rs-fMRI), Blood Oxygen Level Dependent (BOLD) sequence was assessed with the following parameters: 160 volumes, TR = 3000 ms, TE = 30, acquisition matrix 80 × 78, slices = 45, flip angle = 90°, spatial resolution voxel size = 3 × 3 × 3 mm^3^. A 3D T1-weighted sequence was also acquired with the following parameters: TR = 8.1 ms; TE = 3.7 ms, acquisition matrix 240 × 240, slices = 170, flip angle = 8°, resolution voxel size = 1 × 1 × 1 mm^3^.

### Rs-fMRI analysis

**Fig. 1:**
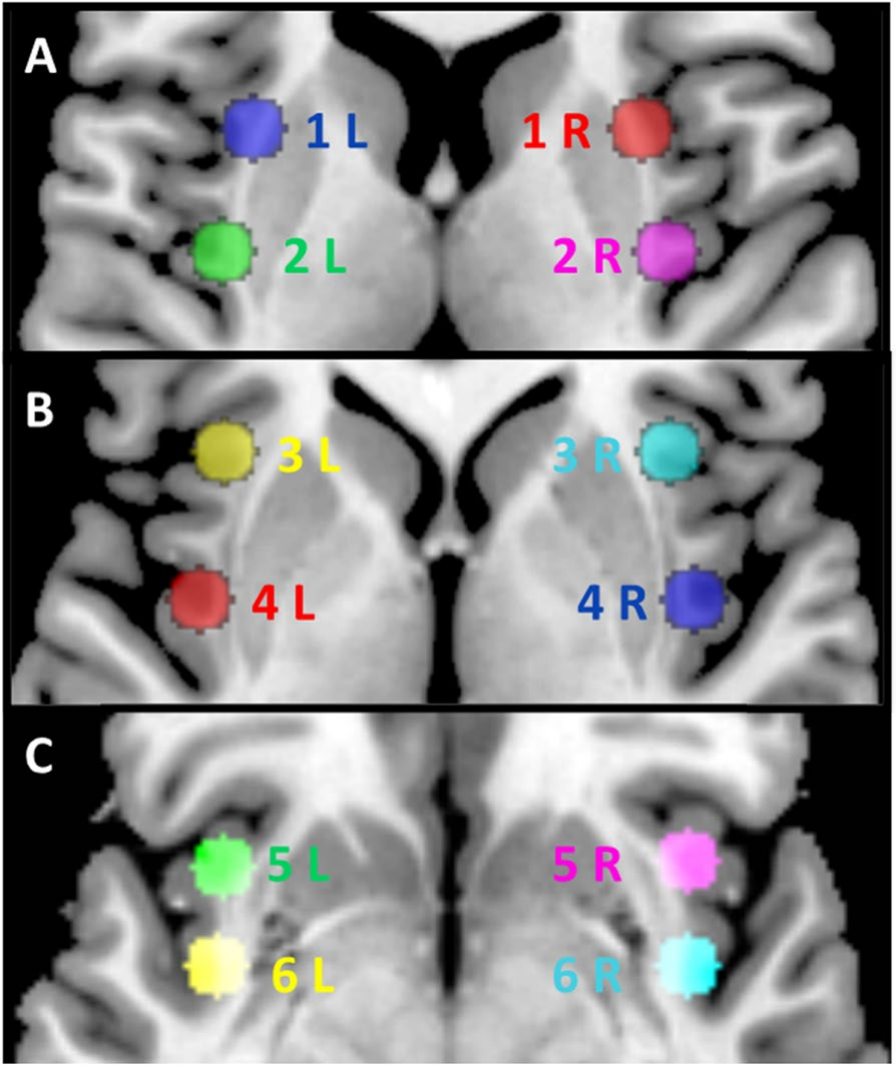
Region of interest (ROI) located in the dorsal (**A**), middle (**B**) and ventral(**C**) insula. These ROI were considered as seeds in the seed-to-voxel analysis. MNI coordinates are given in Table 1

The analysis of the rs-fMRI was processed using Statistical Parametric Mapping (SPM) 12 software (https://www.fil.ion.ucl.ac.uk/spm/), running under MATLAB, and conn toolbox. Pre-processing consisted in spatially realignment, normalization in the Montreal Neurological Institute (MNI) space, smoothing using a 8 mm Gaussian kernel. We performed seed-to-voxels analysis of insular connectivity in MA. The seeds corresponded three Region Of Interest (ROI) dorsal, middle and ventral were placed in the left and right anterior and posterior insular cortex accounting for a total of 12 ROI (Fig. 1). MNI coordinates were derived from *Cauda *et al.,* NeuroImage 2011* [24] and are given in Table 1. We thus conducted a seed-to-voxel analysis between each of these insular ROI, considered as seeds, and the voxels in the whole brain. An average time-course was obtained from the seeds. Correlation maps were generated for each subject in a first level analysis, estimating the correlation coefficient between the whole brain voxels and seeds-time series. The connectivity maps were then introduced in a second level analysis comparing the resting-state FC between patients with MA and HC using a two-sample t-test. The statistical maps were thresholded at *p* < 0.001 and only clusters of more than 10 voxels were retained. Connectivity was assumed significant at *p* < 0.05 corrected for multiple comparisons using the family wise error rate (FWE). A secondary ROI-to-ROI analysis was conducted to precise results of the seed-to-voxels analysis. The average time course was extracted from each ROI and connectivity value was calculated (Fischer Z scores). In ROI-to-ROI analysis, functional coupling was assumed significant at *p* < 0.05. Results were presented with mricron software. Significant results found in MA were afterward compared in patients with MO in order to evaluate the specificity of this result in MA.

**Table 1:** MNI coordinates of the insular ROI (Region of interest)

	Right Number of ROI (x, y, z)	Left Number of ROI (x, y, z)
Antero-dorsal insula	ROI 1 R (31, 12, 8)	ROI 1 L (-31, 12, 8)
Postero-dorsal insula	ROI 2 R (36, -9, 7)	ROI 2 L (-36, -9, 7)
Anterior middle insula	ROI 3 R (36, 19, 1)	ROI 3 L (-36, 19, 1)
Posterior middle insula	ROI 4 R (40, -5, 0)	ROI 4 L (-40, -5, 0)
Antero-ventral insula	ROI 5 R (36, 16, -8)	ROI 5 L (-36, 16, -8)
Postero-ventral insula	ROI 6 R (40, -2, -8)	ROI 6 L (-40, -2, -8)

R right, L Left

### Statistics of clinical data

Qualitative and continuous variables were presented as percentages and arithmetic medians with their corresponding interquartile range. Qualitative variables were compared using the Chi-squared test and continuous variable by using the Wilcoxon-Mann–Whitney test. Correlations between clinical data and the strength of FC, figured as a Z score, were evaluated with the Spearman correlation coefficient. Statistical analyses were performed with the statistical R software (v.4.0.0). All tests were considered significant at the 0.05 level.

### Data availability

Anonymized data not published within this article will be made available on reasonable request from any qualified investigator.

## Results

### Subjects

Two patients with MA and five HC were excluded because of motion artifacts on BOLD sequence. Functional MR images were thus available in 21 patients with MA, 12 patients with MO and 18 HC. The included volunteers had no significant medical history and were similar in age (MA, median age (IQR): 39.0 (12.0) years; MO: 42 (18.7) years; HC: 39 (9.5) years), P > 0.05 and sex (81% women in MA; 75% in MO and 72% in HC), P > 0.05. Seven patients with MA were under preventive therapy (2 betablocker, 2 topiramate, 1 valproic acid, 1 valproic acid and aspirin, 1 oxetorone). All patients with MA had visual aura, 8 of them also had sensory aura and seven dysphasic aura. In MA, median (IQR) duration of migraine was 25 [13] years, annual frequency of attacks was 15 [17] attacks/year and time since last migraine attack was 19 [24] days. No patient with MO was under preventive therapy. In MO, median (IQR) duration of migraine was 21 (10.5) years and monthly migraine days was 3.5 (1.25) days.

### MA vs HC: seed-to-voxel analysis

**Fig. 2:**
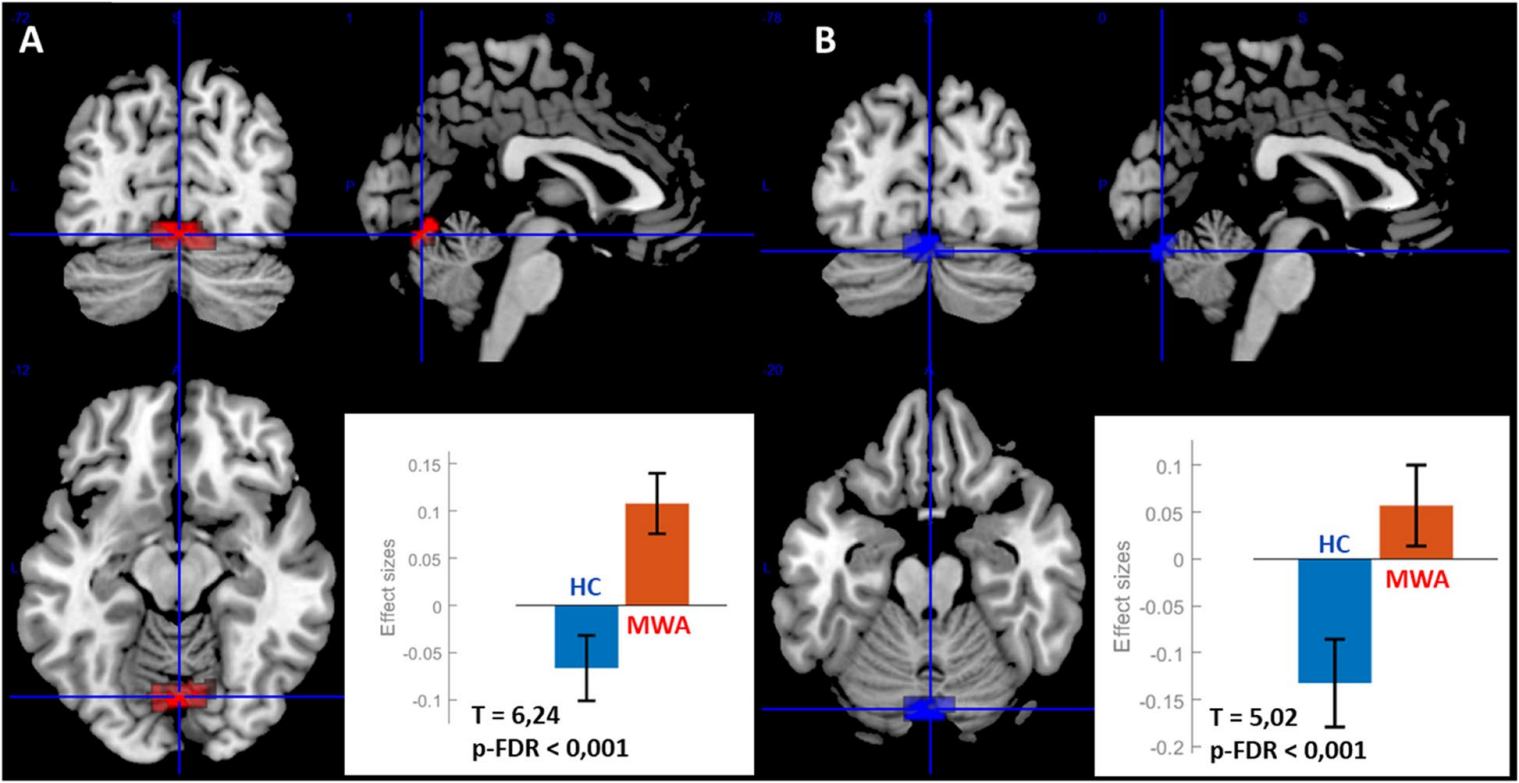
Increased connectivity between ROI 1, antero-dorsal insula, right (**A**) and left (**B**) with cerebellum in MA

**Fig. 3:**
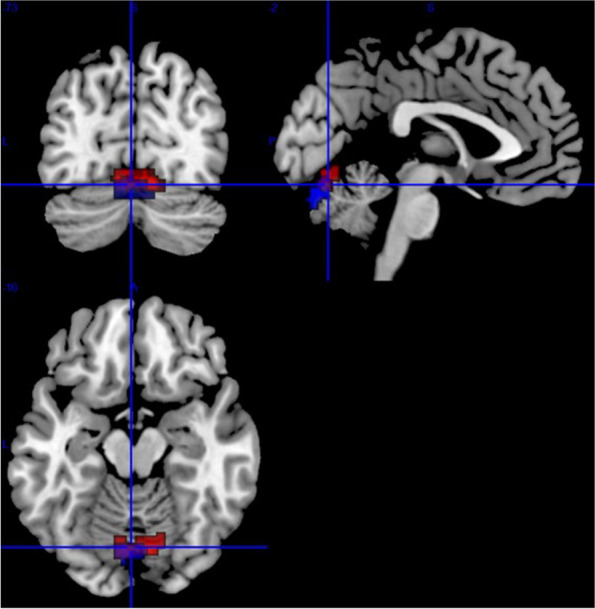
Overlap (purple) of areas highly connected to the right (red) and left (blue) antero-dorsal insula, corresponding to vermis VI

ROI 1 R presented an increased FC in MA with a cluster encompassing vermis 6 (31% volume of the cluster), lingual gyrus left (21%), lingual gyrus right (18%), cerebellum 6 right (18%) and cerebellum 6 left (12%). Size of the cluster: 257, size p-FWE = 0.015, peak p-FWE = 0.043, MNI coordinates (x, y, z) = (06, -72, -12), T = 6,24, p-FDR < 0,001 (Fig. 2). ROI 1 L presented an increased FC in MA with a cluster encompassing cerebellum crus 1 left (20%), vermis 7 (18%), cerebellum Crus 2 left (17%), vermis 6 (13%), cerebellum 6 left (11%), lingual gyrus left (10%), cerebellum Crus 1 right (5%), cerebellum 6 right (2%). Size of the cluster 218, size p-FWE = 0.027, peak p-FWE = 0.806, MNI coordinates (x, y, z) = (-02, -78, -18), T = 5,02, p-FDR < 0,001 (Fig. 2). No significant difference was found between MA and HC for other insular ROI. Then both ROI 1 R and 1 L, corresponding respectively to right and left antero-dorsal insular cortex, presented an increased FC with a cerebellum area. The anatomical labelisation was carried out with SUIT toolbox [25]. The overlapping of the two cerebellar regions connected with the antero-superior insula corresponded to the vermis VI, MNI coordinates (x, y, z) = (00, -76, -17), size of the cluster 86 (Fig. 3).

### MA vs HC: ROI-to-ROI analysis

**Fig. 4:**
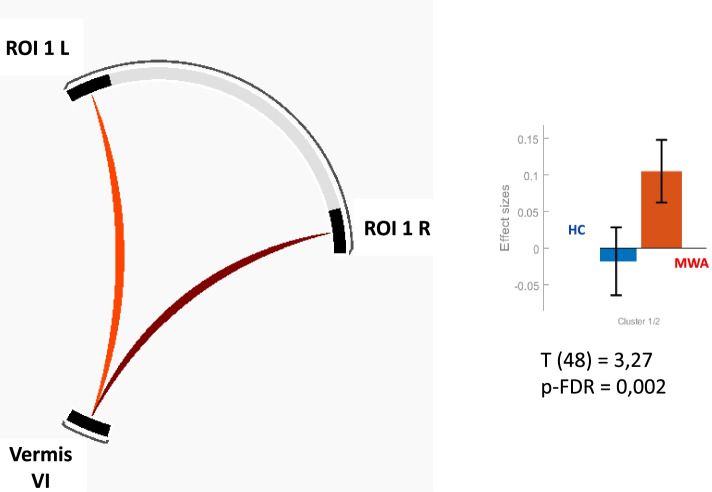
ROI-to-ROI functional coupling of vermis VI with right (ROI 1 R) and left (ROI 1 L) antero-dorsal insula. HC = healthy controls. MA = migraine with aura

Althought the overlap functionally coupled to the antero-dorsal insula corresponded to vermis VI, it is noteworthy that right and left lingual cortices were part of the clusters. As the lingual cortices are involved in MA, we conducted ROI-to-ROI analysis between lingual cortices and insula and between vermis VI and insula. It revealed no FC between the right or the left lingual cortices and ROI 1R or ROI 1L. In contrast, we confirmed the strong FC between the right and left antero-dorsal insula with the vermis VI: T (48) = 3.27; p-FDR = 0.002, Fig. 4.

### Correlation with clinical data in MA

Neither the FC between ROI 1 R and vermis VI nor the FC between ROI 1 L and vermis VI were correlated to clinical features (duration of migraine, frequency of migraine attacks and time since last migraine attack), Table 2.

**Table 2:** Correlation between antero-dorsal insula – vermis VI connectivities and characteristics of migraine (Spearman correlation coefficients)

	Connectivity ROI 1 R – vermis VI (Z score)	Connectivity ROI 1 L – vermis VI (Z score)
Duration of migraine (years)	*ρ* = 0,21; *p* = 0,36	*ρ* = 0,09; *p* = 0,34
Frequency of migraine attack (/year)	*ρ* = -0,40; *p* = 0,08	*ρ* = -0,24; *p* = 0,30
Time since last migraine attack (days)	*ρ* = 0,34; *p* = 0,13	*ρ* = 0,22; *p* = 0,69

### Comparison of MA and HC with Mo by using ROI-to-ROI analysis

We compared the FC of vermis VI to ROI 1R and ROI 1L between MO and HC and between MO and MA. No difference of functional coupling was found between MO and HC. When MA was compared to MO, vermis VI was functionally coupled to ROI 1R but not to ROI 1L, T(48) = 2.96; p-FDR = 0.004.

## Discussion

This explorative study aimed at investigating whether insula exhibits differences in FC in patients with MA using a comprehensive seed-to-voxel analysis of right and left insula with six seeds located within the anterior, posterior, dorsal, middle and ventral insula. We found an increase connectivity of both right and left antero-dorsal insula with the cerebellar vermis VI in patients with MA. This increased FC was not found in patients with MO. Previous studies in migraine have shown that insula exhibits alterations of FC with brain structures involved in pain processing such as thalamus [12], PAG [26, 27], somatosentory cortex [18], or cognitive function such as the default mode network [21, 28] or salience network [17, 20, 21]. In MA specifically, insula exhibited altered FC with occipital cortex and somatosensoriel cortex only in complex MA [17, 18]. However, these findings relied mostly on investigations of other brain areas or partial explorations of insula. To the best of our knowledge, our result is new and its originality could be explained by the methods which consisted of a comprehensive insular FC analysis by distinguishing insula subdivions from each other. Other methodological differences with previous studies should be considered. Previous studies showing an increased FC of the insula with the thalamus involved MO during spontaneous migraine attacks [12], an increased connectivity of insula with the PAG involved patients with allodynia [26, 27], an increased connectivity of insula with the somatosensory cortex involved patients with complex auras [18]. One study showing a lower correlation with the visual cortex set a less conservative threshold of significance for statistical maps [17]. Our study showing an increased FC of both right and left antero-dorsal insula with the cerebellar vermis VI provides a new perpective on the role of insula in MA. Because our study did not demonstrate a correlation between this FC and clinical features of MA, we acknowledge that the clinical significance of our finding is currently undetermined. However, knowledge on the function of the insula and the vermis VI allowed us to postulate two hypotheses to explain this increased FC: one related to the central integration of pain, the other to the control of the parasympathetic autonomic nervous system. Animals and humans studies have highlighted the involvement of the cerebellum in pain [29]. Indeed, studies in healthy volunteers, using fMRI showed an activation of cerebellar lobule VI, VIIIa, crus I and vermal lobule VIIIa evoked by painful stimulation of the left nostril. The cerebellum presented an increased FC with structures involved in pain processing such as rostral pons, PAG, thalamus and cortices regions including insula and face area in the precentral gyrus [30]. Compared to HC, migraine patients presented a higher activation of PAG and left cerebellum crus I in response to nociceptive trigeminal stimulations. Moreover the vermis VI belonged to a cluster that comodulated with migraine-phase [31]. Moreover, functional MR studies revealed cerebellar activation evoked by heat painful stimulations in migraine [32–34]. Although these evidences stressed the role of cerebellum in trigeminal pain processing in migraine, our result did not support clearly a role of vermis-insula FC in pain because this FC was not correlated to migraine feature and was not found in patients with MO. The insula is part of the autonomic nervous system (ANS) network, as well as the anterior cingulate cortex, the pre-frontal cortex and the amygdala [35]. Some studies have suggested that the cerebellum is also involved in the regulation of the cardiovascular ANS. Functional neuroimaging studies in human confirmed that cerebellum is activated during tasks challenging cardiovascular ANS and both anterior insula and vermis cerebellum seem to be involved in the regulation of parasympathetic tone [36–38]. A meta-analysis published in 2013 included studies analysing peripheral signals in response to ANS stimulation by cognitive, affective and somatosensory autonomic nervous system tasks in conjunction with brain imaging in healthy subjects. The ANS response was classified as sympathetic or parasympathetic based on measures of heart rate variability and electrodermal activity. This meta-analysis showed parasympathetic activation of the dorsoanterior insula and vermis VI in addition to other brain structures such as the amygdala or posterior cingulate cortex [38]. The shared role of the cerebellum and insula in the regulation of the cardiovascular ANS is also supported by two activation studies and a connectivity study during ANS stimulation tasks: in a PET study, Critchley et al. have explored the cerebral activation during isometric exercise and mental arithmetic stressor tasks. These tasks induced variation in mean arterial blood pressure (MAP) and heart rate (HR) and were accompanied by an activation of the midline cerebellum, the brainstem in the region of the pontine reticular nuclei and the right dorsal cingulate cortex. In a conjunction analysis, the activation of both the cerebellar vermis and the insular cortex covariated with MAP and HR [39]. Baker et al., in an fMRI study, showed that lower-body negative pressure maneuver (LBNP) induced activation of bilateral insula. The cerebellum and the bilateral insula were activated during the recovery phase of LBNP [40]. A further analysis revealed that vermis VI was part of a cerebellar connectome functionally coupled with bilateral insula, as well as anterior cingulate cortex, bilateral thalamus and bilateral putamen [41]. These previous observations supported the hypothesis that the increased FC between dorsoanterior insula and vermis VI in MA could be related to the cardiovascular parasympathetic ANS control. However, our study was unable to confirm this hypothesis as no recording of cardiovascular parameters was performed during the MR scan. Nevertheless, this hypothesis is likely given the cardiovascular autonomic disorders observed in MA. It has previously been shown that both MA and MO are at increased risk of syncope and orthostatic intolerance [42]. However, one study found that only MA was associated with increased risk of syncope after adjustment on confounders [43]. A review of studies assessing cardiovascular autonomic balance in migraine showed a trend toward greater autonomic dysfunction in MA than in MO, with sympathetic dysfunction being more common than parasympathetic dysfunction [44]. These previous observations supported the hypothesis of ANS-related alteration of FC between dorsoanterior insula and vermis VI in MA. The strengths of our study were the comprehensive exploration of the insula in a seed-to-voxel analysis. The result remained significant after correction for multiple comparisons and was found bilaterally. A random result seemed thus unlikely. ROI-to-ROI analysis excluded the involvement of the lingual cortex in the vicinity and confirmed the functional coupling of the vermis VI with the antero-dorsal insula. Some limitations should be mentioned. The study involved a small sample of volunteers, which may have underestimated differences in connectivity with other brain areas. Although a previous study suggested gender differences of insular connectivity in pain [45], our study sample did not allow for testing gender differences of insular connectivity in migraine. Patients with MO were included from another study previously conducted at our center but the MRI protocol was the same as in the study including patients with MA and HC. Finally, assessment of the cardiovascular ANS during fMRI was not performed which mitigate the interpretation of our result. Further studies are warranted to determine whether the increased functional coupling of antero-dorsal insula with vermis VI reflected an increased parasympathetic tone as we may hypothesize.

## Conclusion

In MA, the bilateral antero-dorsal insula was strongly functionally coupled with the cerebellar vermis IV. Because both regions are involved in the control of the parasympathetic cardiovascular ANS, this functional connectivity could reflect the cardiovascular features of MA. Further research is needed to explore this hypothesis.

## Data Availability

Anonymized data and materials not published within this article will be made available on reasonable request from any qualified investigator.

## Abbreviations

ANS: Autonomic nervous system
BOLD: Blood oxygen level dependent
DMN: Default mode network
FC: Functional connectivity
FEW: Family wise error
fMRI: Functional MRI
HC: Healthy controls
HR: Heart rate
ICHD: International classification of headache disorders
IQR: Interquartile range
LBNP: Lower-body negative pressure maneuver
MAP: Mean arterial blood Pressure
MA: Migraine with Aura
MNI: Montreal neurological institute
MO: Migraine without aura
MRI: Magnetic resonance imaging
PAG: PeriAqueductal grey matter
PET: Positron tomography emission
ROI: Region of Interest
SPM: Statistical parametric mapping
Rs-fMRI: Resting functional MRI
